# Cutaneous manifestations as the presenting signs of diffuse large B-cell lymphoma/high-grade B-cell lymphoma with MYC and BCL2 rearrangements

**DOI:** 10.1016/j.jdcr.2024.09.032

**Published:** 2024-11-23

**Authors:** Gabriela Piñero-Crespo, Alison H. Kucharik, Wei-Shen Chen, Dietrich Werner Idiáquez, Josef Doenges, Yumeng Zhang, Lubomir Sokol, Frank Glass

**Affiliations:** a College of Medicine, University of South Florida Morsani, Tampa, Florida; b Department of Dermatology and Cutaneous Surgery, University of South Florida Morsani College of Medicine, Tampa, Florida; c Department of Pathology – Hematopathology, Moffitt Cancer Center, Tampa, Florida; d Cutaneous Lymphoma Multidisciplinary Division, Moffitt Cancer Center, Tampa, Florida

**Keywords:** cutaneous diffuse large B-cell lymphoma, high-grade B-cell lymphoma

## Introduction

Non-Hodgkin’s lymphoma is one of the most common hematologic malignancies worldwide, with aggressive diffuse large B-cell lymphoma (DLBCL) being one of the most common subtypes. The family of DLBCL can be further subdivided into specific entities defined by the World Health Organization (WHO) depending on morphology, genetic features, viral association, site of origin, or other criteria.^1^ Subclassification into these entities is important to evaluate prognosis and treatment options, as some subtypes, such as diffuse large B-cell lymphoma/high-grade B-cell lymphoma with MYC and BCL2 rearrangements (DLBCL/HGBL-MYC/BCL2), have worse prognosis and require alternative treatment regimens.^1^

DLBCL/HGBL-MYC/BCL2 was defined as a distinct entity by the fifth edition of the WHO classification of hematolymphoid tumors in 2023 and is associated with advanced disease and extranodal involvement of the bone marrow and central nervous system (CNS).^1^ Approximately 10% of DLBCL cases have MYC rearrangements, and 40% of those constitute the DLBCL/HGBL-MYC/BCL2 subtype.^1^

Associated risk factors for DLBCL in general include prior radiation treatment, obesity, and smoking.^2^ Extranodal involvement occurs in approximately 40% of cases, but cutaneous involvement is rare. When skin is involved, lesions commonly present as plaques, papules, small nodules, or ulcers.^3^ This report highlights cutaneous manifestations as the unusual presenting signs of DLBCL/HGBL-MYC/BCL2.

## Case

**Fig 1:**
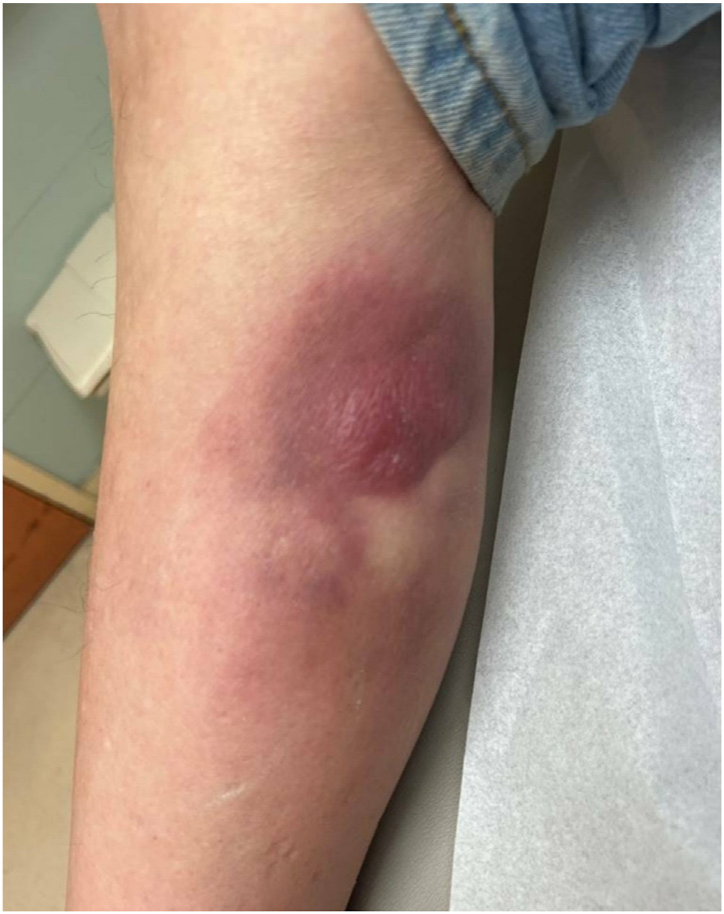
Cutaneous diffuse large B-cell lymphoma/high-grade B-cell lymphoma with MYC and BCL2 rearrangements lesion on the lower portion of the leg at initial presentation at the emergency department.

A 65-year-old Hispanic man with a past medical history of chronic obstructive pulmonary disease, coronary artery disease, hypertension, 48 pack-year smoking history, and chronic hyponatremia due to possible syndrome of inappropriate antidiuretic hormone secretion, presented to the emergency department for worsening “bug bites,” edema, and paresthesia in the lower extremities (Fig 1). Further workup revealed hyponatremia, for which he was admitted, and subsequently treated with antibiotics for presumed cellulitis from his reported bug bites. Because of his history of hypertension, reported orthopnea, and edema, he underwent a negative congestive heart failure work up, and a negative deep vein thrombosis screening. With improvement of his hyponatremia, “bug bites,” and edema, he was discharged 4 days later.

**Fig 2:**
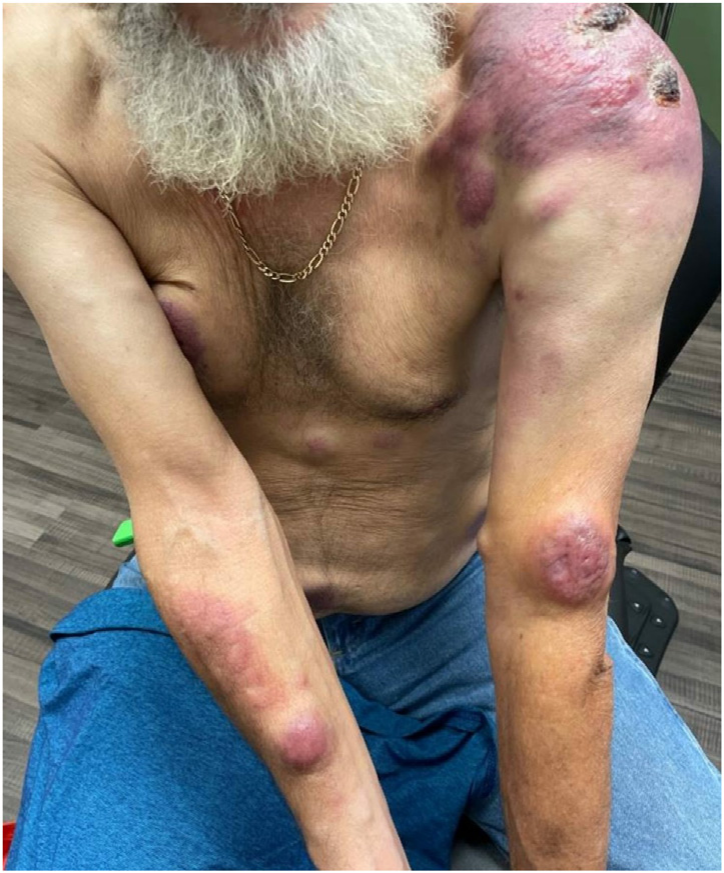
Cutaneous diffuse large B-cell lymphoma/high-grade B-cell lymphoma with MYC and BCL2 rearrangements presentation at outpatient dermatology clinic, a month after initial emergency department presentation.

**Fig 3:**
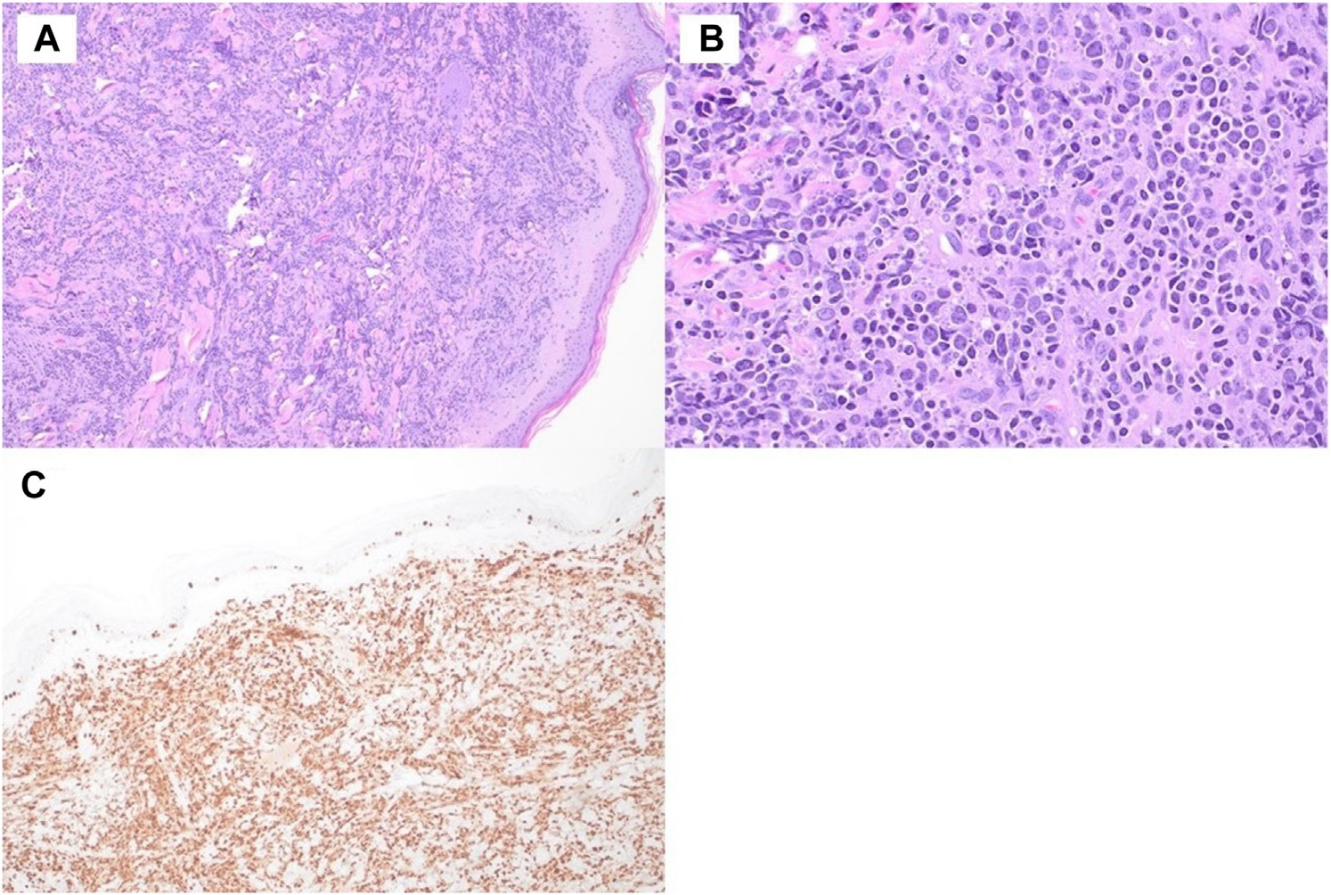
**A,** Low-power view histopathology of skin biopsy revealing dermal infiltrate of atypical lymphoid cells, consistent with cutaneous diffuse large B-cell lymphoma/high-grade B-cell lymphoma (DLBCL/HGBCL). **B,** High power view of cutaneous DLBCL/HGBCL histopathology. **C,** Cutaneous DLBCL/HGBCL histopathology with immunohistochemistry stain positive for Ki-67, indicating high proliferation.

Within 1 week, the patient experienced unilateral Bell palsy and 2 firm, mobile, painless nodules on the trunk and arm. One month after the initial presentation, the patient presented at the outpatient dermatology clinic with 19 tender, skin-colored to red/violaceous plaques and nodules, ranging from 1 to 19 cm in size, located on the trunk and all 4 extremities (Fig 2). Review of systems at this time was positive for congestion, sinus pressure, and subjective fevers. Three biopsies revealed dermal infiltrate of atypical lymphoid cells, which was consistent with DLBCL/high-grade B-cell lymphoma (Fig 3, *A*, *B*). Ki-67 stain (90%) revealed a high proliferation rate (Fig 3, *C*). Immunohistochemistry was also positive for: CD20, CD19, CD10, c-Myc, and Bcl-2. Fluorescent in situ hybridization was positive for c-MYC and BCL2 gene rearrangements, confirming the diagnosis as DLBCL/HGBL-MYC/BCL2.

Further oncologic workup showed mildly hypocellular bone marrow (20%) with low level involvement (<5%) of DLBCL/HGBL-MYC/BCL2. Flow cytometry of peripheral blood also showed low level involvement with B-cell lymphoma, CD5 dim positive and CD10 positive. Gene rearrangement studies showed monoclonal Ig heavy chain and light chain rearrangements, with no monoclonal T-cell receptor rearrangement. Imaging workup revealed diffuse lymphadenopathy, pulmonary infiltrates favored to represent lymphoma, and maxillary sinus/ethmoid air cell involvement.

**Fig 4:**
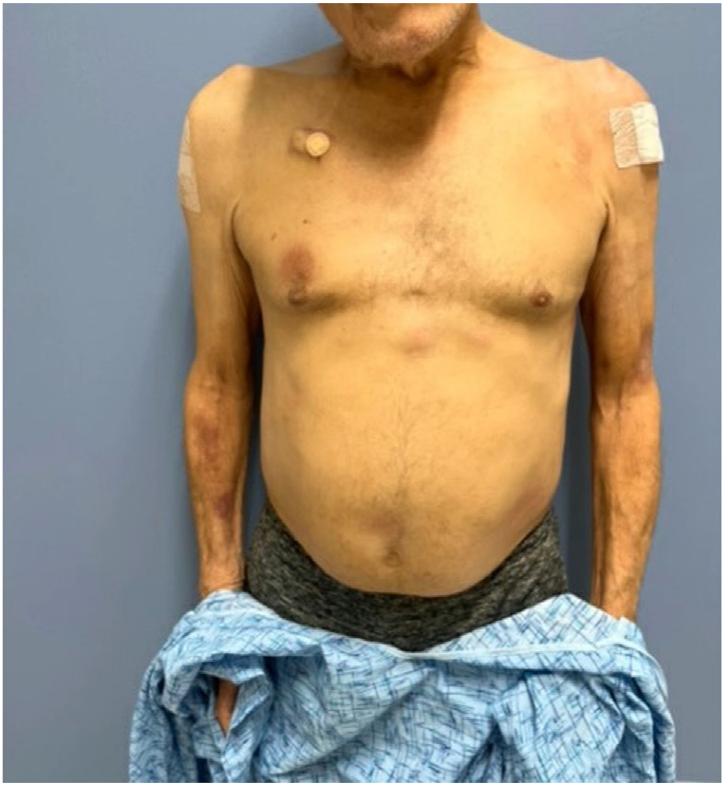
Almost complete resolution of cutaneous lesions of diffuse large B-cell lymphoma/high-grade B-cell lymphoma with MYC and BCL2 rearrangements 1 month after initiating chemotherapy.

He began treatment and underwent 1 cycle of dose-adjusted etoposide, prednisone, vincristine, cyclophosphamide, and doxorubicin with intrathecal methotrexate, 1 cycle of rituximab, and 1 cycle of high-dose methotrexate. Lumbar puncture revealed CNS involvement, so the treatment regimen was changed to favor stronger CNS penetration. He subsequently began treatment with hyperfractionated cyclophosphamide, vincristine, doxorubicin, and dexamethasone (HyperCVAD) with intrathecal methotrexate and cytarabine. At 1 month into treatment, we observed an almost complete response of his cutaneous lesions (Fig 4), with sparse postinflammatory hyperpigmentation. Four months into treatment, imaging revealed no evidence of disease. After completion of 8 cycles of HyperCVAD, full body positron emission tomography computed tomography and bone marrow biopsy demonstrated complete metabolic response. He continues to follow up with Hematology-Oncology for surveillance and is being considered for future stem cell transplant or chimeric antigen receptor T-cell therapy.

## Discussion

Although cutaneous involvement of DLBCL is rare, it is important to recognize it early because of its rapid progression. This unusual presentation as lower extremity “bug-bites,” paresthesia, and edema along with systemic B symptoms may elicit a broad differential, including cellulitis, allergic reaction, or venomous insect bite. Further workup with skin biopsy would be warranted in patients that do not respond to antibiotics or antihistamines and have a rapid progression such as our patient. Systemic B symptoms and rapid cutaneous involvement of violaceous nodules and plaques should alert dermatologists and oncologists that a systemic lymphoma with cutaneous involvement may be likely. The initial presentation of skin lesions on the lower extremity may be reminiscent of the variant named primary cutaneous DLBCL, leg type. However, given the extensive systemic involvement and pathology findings in this case, the diagnosis was favored to be systemic DLBCL/HGBL-MYC/BCL2 with cutaneous manifestations, rather than primary cutaneous DLBCL, leg type with extracutaneous manifestations.^4^

Our patient also experienced a unilateral Bell palsy. Rarely, DLBCL has been associated Guillain-Barré syndrome^5^ and cranial nerve involvement^6^ in the literature. Facial hemiplegia and paresthesia may precede cutaneous DLBCL, providing a subtle yet important clue for diagnosis.

Pathology and genetic analyses are crucial for diagnosis and prognostic assessment. Double-expressor phenotypes, overexpressing both MYC and BCL2, and rearrangements in these genes are associated with poor prognosis and poor response to traditional rituximab, cyclophosphamide, doxorubicin, vincristine, and prednisone chemotherapy.^7^ These high-risk mutations have been shown to have improved survival benefits with dose-adjusted etoposide, prednisone, vincristine, cyclophosphamide, and doxorubicin.^8^ However, HyperCVAD therapy may be more suitable for CNS involvement. This regimen may have higher complete response rates, but this must be weighed against higher complication risk.^9^

## Conflicts of Interest

None disclosed.
